# Supplementary material for: A durable and pH-universal self-standing MoC–Mo2C heterojunction electrode for efficient hydrogen evolution reaction
Source: Nat Commun. 2021 Nov 22;12:6776. doi: 10.1038/s41467-021-27118-6 (PMC8608917; doi:10.1038/s41467-021-27118-6)
Supplement: Supplementary file 1 — Supplementary Information [file 41467_2021_27118_MOESM1_ESM.pdf]

Supplementary Information for

A durable and pH-universal self-standing MoC-Mo_2C heterojunction electrode for efficient hydrogen evolution reaction

Wei Liu[1, 2], Xiting Wang[3], Fan Wang[1, 2], Kaifa Du[1, 2], Zhaofu Zhang[4], Yuzheng Guo[3]*, Huayi Yin[1, 2]* and Dihua Wang[1, 2, 5]*

1. School of Resource and Environmental Science, Wuhan University, Wuhan 430072, China

2. International Cooperation Base for Sustainable Utilization of Resources and Energy in Hubei Province, Wuhan University, Wuhan 430072, China

3. School of Electrical Engineering and Automation, Wuhan University, Wuhan 430072, China

4. Department of Engineering, University of Cambridge, Cambridge, CB2 1PZ, United Kingdom.

5. State Key Laboratory of Water Resources and Hydropower Engineering Science, Wuhan University, Wuhan 430072, China

***Corresponding author**:

Prof. Dihua Wang (wangdh@whu.edu.cn)

Prof. Huayi Yin (yinhuayi@whu.edu.cn);

Prof. Yuzheng Guo (yguo@whu.edu.cn);

Supplementary information contains the following contents:

1. Supplementary Methods………………………………………………………………. 3

2. ECSA calculation…………………………………………………………………… 4

3. TOF calculation……………………………………………………………………….. 4

4. Supplementary Tables…………………………………………………………….... 8

5. Supplementary Figures…………………………………………………………. 15

6. Supplementary References…………………………………………………………... 35

1. Supplementary Methods

The mechanism of the electrodeposition of Mo carbide films

The MoC-Mo_2C electrode was prepared by one-step electrochemical surface engineering by constant-current electrolysis (3.5 mA cm^{-2} for only 2 h) with continuous injection of CO_2 into molten Li_2CO_3-K_2CO_3 salts. As shown in Figure 1a in manuscript, the injected CO_2 was firstly captured by O^{2-} to form soluble CO_3^{2-} ($CO_2 + O^{2-} = CO_3^{2-}$) [Energy Environ. Sci., 2013, 6, 1538-1545]. Then, CO_3^{2-} got electrons and were reduced to carbon atoms ($CO_3^{2-} + 4e^- = C + 3O^{2-}$) on the surface of the molybdenum (Mo) plate. At the same time, the released O^{2-} diffused to the inert anode and discharged to O_2. Under the synergy of high temperature molten salts and electric field, the deposited carbon diffused into the interior of the Mo plate and reacted with Mo atoms spontaneously to generate MoC/Mo_2C (C + Mo = MoC, $\Delta G_{T=500\text{-}900℃}$=-29.5 kJ/mol～-30.6 kJ/mol; C + 2Mo = Mo_2C, $\Delta G_{T=500\text{-}900℃}$=-47.3 kJ/mol～-45.7 kJ/mol), forming the MoC-Mo_2C HER electrode finally.

In previous work, most Mo_xC films were prepared using a hydrothermal method by which molybdates were used as the Mo precursor along with a reducing agent to drive the formation of carbides. In molten salts, the deposition of carbide was usually conducted in molten halides containing molybdates and carbonates ions as the Mo and C feedstock. However, all electrolyzers used graphite as the anode because it is difficult to employ a low-cost inert anode in molten halides. In previous work, the aim is to prepare a protective coating rather than a catalytic layer.

2. ECSA calculation:

The electrochemical active surface area (ECSA) can be estimated using the double-layer capacitances (C_{dl}). The specific capacitance for a flat surface is supposed to be ~ 40 μF cm^{-2}, and the ECSA is estimated by the following formula:

$$\text{ECSA} = \frac{Specific\ capacitance\ (\mu F\ cm^{-2})}{40\ \mu F\ cm^{-2}\ per\ cm^2_{ECSA}} \qquad (1)$$

3. TOF calculation:

TOF values are calculated according to the method reported by the Jaramillo group [S41-S42]. Detailed process is shown as follows:

To calculate the per-site TOF, we use the following formula:

$$\text{TOF} = \frac{\#\ number\ of\ total\ hydrogen\ turnovers/cm^2}{\#\ number\ of\ active\ of\ sites/cm^2} \qquad (2)$$

The total number of hydrogen turnovers is calculated from the current density according to:

$$\text{No. of H}_2 = (j\frac{mA}{cm^2})(\frac{1C\ s^{-1}}{1000\ mA})(\frac{1\ mol\ e^{-1}}{96485.3\ C})(\frac{1\ mol\ H_2}{2\ mol\ e^{-1}})(\frac{6.022\times10^{23} H_2 mulecules}{1\ mol\ H_2}) = 3.12 \times 10^{15}\frac{H_2/s}{cm^2}\ per\frac{mA}{cm^2}$$

The active sites per real surface area are calculated from the following formula:

$$\text{No. of active sites} = (\frac{No.of\ atoms/unit\ cell}{Volume/unit\ cell})^{\frac{2}{3}}$$

Based on the above-mentioned results, the TOF value can be described as follows:

$$\text{TOF} = \frac{(3.12\times10^{15}\frac{H_2/s}{cm^2}per\frac{mA}{cm^2})\times j}{no.of\ active\ sites\times \text{ECSA}}$$

It is noteworthy that both MoC-Mo_2C-690 and MoC-Mo_2C-790 electrodes are composed of MoC and Mo_2C, so the content of MoC and Mo_2C in the MoC-Mo_2C-690 and MoC-Mo_2C-790 electrode can be used to calculate the number of active sites. Here, we employ the XRD patterns of the MoC-Mo_2C-690 and MoC-Mo_2C-790 electrodes to estimate the content of MoC

and Mo_2C using the area ratio of the two substances.

Thus, the calculation of the number of active sites of MoC-Mo_2C-690 and MoC-Mo_2C-790 should follow the formula:

$$\text{No. of active site} \times \text{ECSA} = \left(\frac{No.of\ atoms\ (MoC)/unit\ cell}{Volume(MoC)/unit\ cell}\right)^{\frac{2}{3}} \times \text{x} \times \text{ECSA} + \left(\frac{No.of\ atoms\ (Mo_2C)/unit\ cell}{Volume(Mo_2C)/unit\ cell}\right)^{\frac{2}{3}} \times \text{y} \times \text{ECSA}$$

Where x and y are the molar ratio of MoC and Mo_2C at the surface of MoC-Mo_2C-690 and MoC-Mo_2C-790 electrodes.

As it is known that the unit cell of Mo contains two atoms with a volume of 31.1 Å^3, MoC contains one Mo atom and one C atom with a volume of 81.7 Å^3 and Mo_2C contains two Mo atoms and one C atom with a volume of 148.7 Å^3. From the phase composition ratio of MoC and Mo_2C determined by the XRD phase quantitative analysis using Jade 6.5, we can estimate that MoC-Mo_2C-690 contains 91.4% MoC and 8.6% Mo_2C and MoC-Mo_2C-790 contains 65.4% MoC and 34.6% Mo_2C.

Thus,

For Mo/C-590:

$$\text{No. of active sites} = \left(\frac{No.of\ atoms/unit\ cell}{Volume/unit\ cell}\right)^{\frac{2}{3}}$$

$$\text{No. of active sites} = \left(\frac{2\ atoms/unit\ cell}{31.1\text{Å}^3/unit\ cell}\right)^{\frac{2}{3}}$$

$$\text{No. of active sites} = 1.6 \times 10^{15}\ \text{atoms cm}^{-2}$$

$$\text{ECSA} = \frac{Specific\ capacitance\ (\mu F\ cm^{-2})}{40\ \mu F\ cm^{-2}\ per\ cm^2_{ECSA}} = \frac{5680\ (\mu F\ cm^{-2})}{40\ \mu F\ cm^{-2}\ per\ cm^2_{ECSA}}$$

$$\text{TOF} = \frac{(3.12 \times 10^{15}\frac{H_2/s}{cm^2}\ per\frac{mA}{cm^2}) \times j}{no.of\ active\ sites \times ECSA}$$

For MoC-Mo_2C-690:

$$\text{No. of active sites} = (\frac{No.of\ atoms/unit\ cell}{Volume/unit\ cell})^{\frac{2}{3}}$$

$$\text{No. of active sites (MoC)} = (\frac{2\ atoms/unit\ cell}{81.7Å^3/unit\ cell})^{\frac{2}{3}}$$

No. of active sites (MoC) =8.45×10^{14} atoms cm^{-2}

$$\text{No. of active sites } (Mo_2C) = (\frac{3\ atoms/unit\ cell}{148.7Å^3/unit\ cell})^{\frac{2}{3}}$$

No. of active sites (Mo_2C) =7.41×10^{14} atoms cm^{-2}

$$\text{ECSA} = \frac{Specific\ capacitance\ (\mu F\ cm^{-2})}{40\ \mu F\ cm^{-2}\ per\ cm^2_{ECSA}} = \frac{28250(\mu F\ cm^{-2})}{40\ \mu F\ cm^{-2}\ per\ cm^2_{ECSA}}$$

$$\text{TOF} = \frac{(3.12 \times 10^{15} \frac{H_2/s}{cm^2}\ per \frac{mA}{cm^2}) \times j}{no.of\ active\ sites(MoC) \times x \times ECSA + no.of\ active\ sites(Mo_2C) \times y \times ECSA}$$

Where x=91.4% and y=8.6%.

For MoC-Mo_2C-790:

$$\text{No. of active sites} = (\frac{No.of\ atoms/unit\ cell}{Volume/unit\ cell})^{\frac{2}{3}}$$

$$\text{No. of active sites (MoC)} = (\frac{2\ atoms/unit\ cell}{81.7Å^3/unit\ cell})^{\frac{2}{3}}$$

No. of active sites (MoC) =8.45×10^{14} atoms cm^{-2}

$$\text{No. of active sites } (Mo_2C) = (\frac{3\ atoms/unit\ cell}{148.7Å^3/unit\ cell})^{\frac{2}{3}}$$

No. of active sites (Mo_2C) =7.41×10^{14} atoms cm^{-2}

$$\text{ECSA} = \frac{Specific\ capacitance\ (\mu F\ cm^{-2})}{40\ \mu F\ cm^{-2}\ per\ cm^2_{ECSA}} = \frac{111120(\mu F\ cm^{-2})}{40\ \mu F\ cm^{-2}\ per\ cm^2_{ECSA}}$$

$$\text{TOF} = \frac{(3.12 \times 10^{15} \frac{H_2/s}{cm^2}\ per \frac{mA}{cm^2}) \times j}{no.of\ active\ sites(MoC) \times x \times ECSA + no.of\ active\ sites(Mo_2C) \times y \times ECSA}$$

Where x=65.4% and y=34.6%

For Mo_2C-890:

$$\text{No. of active sites} = (\frac{No.of\ atoms/unit\ cell}{Volume/unit\ cell})^{\frac{2}{3}}$$

$$\text{No. of active sites} = (\frac{3\ atoms/unit\ cell}{148.7Å^3/unit\ cell})^{\frac{2}{3}}$$

No. of active sites = 7.41×10^{14} atoms cm^{-2}

$$\text{ECSA} = \frac{Specific\ capacitance\ (\mu F\ cm^{-2})}{40\ \mu F\ cm^{-2}\ per\ cm^2_{ECSA}} = \frac{34120\ (\mu F\ cm^{-2})}{40\ \mu F\ cm^{-2}\ per\ cm^2_{ECSA}}$$

$$\mathrm{TOF} = \frac{(3.12 \times 10^{15} \frac{H_2/s}{cm^2}\ per \frac{mA}{cm^2}) \times j}{no.of\ active\ sites \times \mathrm{ECSA}}$$

4. Supplementary Tables

Supplementary Table 1. Summary of HER activities of different electrodes in 0.5M H_2SO_4.

Electrodes	*η10* [a] (mV)	*η100* [b] (mV)	*η500* [c] (mV)	Tafel slope [d] (mV dec^{-1})	j_0 [e] (mA cm^{-2})	R_{ct} [f] (Ω)
**Pt**	61.8	123	352	35	1.54×10^{-1}	-
**Mo/C-590**	268	401	580	103	3.34×10^{-2}	107.6
**MoC-Mo$_2$C-690**	159	270	362	80	1.05×10^{-1}	7.06
**MoC-Mo$_2$C-790**	114	183	256	62	1.49×10^{-1}	2.69
**Mo$_2$C-890**	222	316	427	85	5.57×10^{-2}	23.26

a. Overpotential (V vs.RHE) at the current density of 10 mA cm^{-2}.

b. Overpotential (V vs.RHE) at the current density of 100 mA cm^{-2}.

c. Overpotential (V vs.RHE) at the current density of 500 mA cm^{-2}.

d. Exchange current densities obtained from Tafel plots.

e. Exchanged current densities.

f. Charge transfer resistance obtained from EIS at the overpotential of 130mV.

Supplementary Table 2. Comparison of HER performances in 0.5M H_2SO_4 for MoC-Mo_2C-790 with other self-supported electrodes.

HER catalyst	Electrolytes	η10 (mV)	Tafel slope (mV dec^{-1})	$j_{\eta=250}$ (mA cm^{-2})	Reference
**MoC-Mo_2C-790**	0.5M H_2SO_4	114	62	447	This work
**2D MoS_2**	0.5M H_2SO_4	143	71	50	Nano Energy, 2019. [S39]
**1D-DRHA MoS_2**	0.5M H_2SO_4	119	50.7	≈70	Applied Catalysis B: Environmental, 2019. [S27]
**S-MoS_2@C**	0.5M H_2SO_4	136	78	-	Advanced Energy Materials, 2019.[S46]
**MoS_2 ML**	0.5M H_2SO_4	126	67	≈280	Advanced Energy Materials, 2018. [S17]
**r mPF-MoS_2**	0.5M H_2SO_4	210	90	≈50	Nature communications, 2017. [S26]
**Mo_2C/CLCN**	0.5M H_2SO_4	145	48.5	≈100	Nano Energy, 2017. [S18]
**P-MoO_{3-x}-6**	0.5M H_2SO_4	166	42	≈80	Small, 2017. [S19]
**MoS_2/CC**	0.5M H_2SO_4	150	50	≈86	Journal of Materials Chemistry A, 2015. [S20]
**ce-MoS_2**	0.5M H_2SO_4	191	64	≈100	Advanced Materials, 2017. [S21]
**FLNPC@MoPNC/MoP-C/CC**	0.5M H_2SO_4	74	50	≈175	Advanced Functional Materials, 2018. [S22]
**MoP-CA2**	0.5M H_2SO_4	125	54	≈245	Advanced Materials, 2014. [S23]
**α-MoB_2**	0.5M H_2SO_4	149	75.8	≈250	Journal of the American Chemical Society, 2017. [S24]
**3D N-graphene-C_3N_4**	0.5M H_2SO_4	75	49.1	≈60	ACS nano, 2015. [S25]

Supplementary Table 3. Comparison of HER performances in 1M KOH for the MoC-Mo_2C-790 electrode with other self-supported electrodes.

HER catalyst	Electrolytes	η10 (mV)	Tafel slope (mV dec^{-1})	$j_{\eta=250}$ (mA cm^{-2})	Reference
**MoC-Mo_2C-790**	1M KOH	98.2	59	330	This work
**$(Gd_{0.5}La_{0.5})BaCo_2O_{5.5+\delta}$**	1M KOH	210	35.5	≈350	Nature communications, 2019. [S28]
**Ni–Mo–N/CFC**	1M KOH	40	70	≈140	Nature communications, 2019. [S13]
**A-CFC**	1M KOH	71	54.5	≈125	Nature communications, 2019.[S43]
**S-MoS_2@C**	1M KOH	155	164	≈60	Advanced Energy Materials, 2019.[S46]
**G-$Co_{0.6}Fe_{0.4}$**	1M KOH	150	-	≈20	Advanced Energy Materials, 2019. [S44]
**Cu_1Ni_2-N**	1M KOH	71.4	106.5	≈200	Advanced Energy Materials, 2019.[S45]
**MoP/rGO**	1M KOH	150	51	≈80	Chemical Communications, 2016. [S29]
**Fe-Ni_2P**	1M KOH	100	56	≈75	Advanced Functional Materials, 2017.[S30]
**$NiCo_2S_4$ /Ni_3S_2 /NF**	1M KOH	119	105.2	≈120	ACS applied materials & interfaces. [S31]
**Co-Ni-B/CC**	1M KOH	80	88.2	≈100	Electrochemistry Communications. [S32]
**MoP_2 NPs/Mo**	1M KOH	194	80	≈95	Nanoscale 2016. [S33]
**Mo_2C-C**	1M KOH	136	66	≈50	Nano Energy, 2017. [S34]
**NiO NRs-m-Ov**	1M KOH	110	100	≈75	Nano Energy, 2018. [S35]
**NiFeOx/CFP**	1M KOH	145	34.2	≈100	Nat. Commun., 2015. [S36]

Supplementary Table 4. Survey of the HER stability of representative electrocatalysts in 0.5M H_2SO_4.

Electrolycatalyst	Electrolyte	Current density	Time	Reference
$MoC-Mo_2C$-790	0.5M H_2SO_4	34mA cm^{-2}	2000 h	This work
	0.5M H_2SO_4	100 mA cm^{-2}	100h	
	0.5M H_2SO_4	300 mA cm^{-2}	50h	
	0.5M H_2SO_4	500 mA cm^{-2}	50h	
$WS_2/Ni_5P_4-Ni_2P$	0.5M H_2SO_4	10 mA cm^{-2}	22h	Nano Energy, 2019 [S2]
A-CFC	0.5M H_2SO_4	10 mA cm^{-2}	30 h	Nature communications, 2019. [S43]
W-CoP NAs/CC	0.5M H_2SO_4	10 mA cm^{-2}	36 h	Small, 2019. [S37]
S-MoS_2@C	0.5M H_2SO_4	10 mA cm^{-2}	24 h	Advanced Energy Materials, 2019. [S46]
MoB/g-C3N4	0.5M H_2SO_4	10 mA cm^{-2}	48 h	Angewandte Chemie International Edition, 2018. [S38]
AgNi-5 NCs	0.5M H_2SO_4	15 mA cm^{-2}	120 h	Angewandte Chemie International Edition, 2019. [S40]
ce-MoS2	0.5M H_2SO_4	10 mA cm^{-2}	130 h	Advanced Materials, 2017. [S15]
FLNPC@MoP-NC/MoP-C/CC	0.5M H_2SO_4	10 mA cm^{-2}	60 h	Advanced Functional Materials, 2018. [S16]
MoS_{2+x}/N-CNTs/CP	0.5M H_2SO_4	≈11 mA cm^{-2}	1 h	Advanced Materials, 2017. [S5]
$MoS_xSe_yP_z$	0.5M H_2SO_4	11 mA cm^{-2}	10 h	Small, 2018. [S8]
1D-DRHA MoS2	0.5M H_2SO_4	10 mA cm^{-2}	250 h	Applied Catalysis B: Environmental, 2019. [S9]
$W_{0.5}Mo_{0.5}S_2$	0.5M H_2SO_4	10 mA cm^{-2}	10 h	ACS Catalysis, 2018. [S10]
monolayer MoS_2	0.5M H_2SO_4	20 mA cm^{-2}	200 h	Nature communications, 2019. [S11]

Supplementary Table 5. Survey of the HER stability of representative electrocatalysts in 1 M KOH.

Electrolycatalyst	Electrolyte	Current density	Time	Reference
**MoC-Mo_2C-790**	1M KOH	100 mA cm^{-2}	100 h	This work
	1M KOH	300 mA cm^{-2}	50 h	
	1M KOH	500 mA cm^{-2}	50 h	
**Ni–Mo–N/CFC**	1M KOH	10 mA cm^{-2}	12 h	Nature communications, 2019. [S13]
**$NiFeO_x$/CFP**	1M KOH	10 mA cm^{-2}	100 h	Nature communications, 2015. [S12]
**$NiCo_2S_4$**	1M KOH	10 mA cm^{-2}	1000 h	Nature communications, 2018. [S14]
**$(Ni_{0.33}Fe_{0.67})_2P$**	1M KOH	165 mA cm^{-2}	12 h	Advanced Functional Materials, 2017. [S1]
**TiN@Ni_3N nanowires**	1M KOH	35 mA cm^{-2}	10h	Journal of Materials Chemistry A, 2016.[S3]
**BCF/Mo_2C**	1M KOH	≈25 mA cm^{-2}	50 h	ACS applied materials & interfaces, 2017.[S4]
**$(Gd_{0.5}La_{0.5})BaCo_2O_{5.5+\delta}$**	1M KOH	50 mA cm^{-2}	10 h	Advanced Functional Materials, 2016. [S6]
**S-MoS_2@C**	1M KOH	10 mA cm^{-2}	24 h	Advanced Energy Materials, 2019. [S46]
**$Co_{0.31}Mo_{1.69}C$/MXene/NC**	1M KOH	10 mA cm^{-2}	100 h	Advanced Energy Materials, 2019. [S7]

Supplementary Table 6. Fitting parameters (peak position, peak area and species percentage) for both Mo $3d_{5/2}$ and Mo $3d_{3/2}$ spectra taken on electrolytic electrodes prepared at 590 ºC, 690 ºC, 790 ºC and 890 ºC.

Samples	Species	B.E.(ev)	B.E.(ev)	Area	Area	Mo^{3+}/Mo^{2+}	Mo^{2+} and Mo^{3+} (%)
		$3d_{5/2}$	$3d_{3/2}$	$3d_{5/2}$	$3d_{3/2}$		
**590** ºC	Mo^0	227.6	230.8	1660	960	1.02	12.6
	Mo^{2+}	228.2	231.3	499	420		
	Mo^{3+}	228.8	231.9	510	430		
	Mo^{4+}	229.9	233.0	320	150		
	Mo^{6+}	232.5	235.5	1161	920		
**690** ºC	Mo^{2+}	228.2	231.3	17885	9700	1.98	68.6
	Mo^{3+}	228.8	231.9	29005	25285		
	Mo^{4+}	229.9	233.0	11085	9985		
	Mo^{6+}	232.5	235.5	12600	9800		
**790** ºC	Mo^{2+}	228.2	231.3	35437	21049	1.42	73.5
	Mo^{3+}	228.8	231.9	46402	33052		
	Mo^{4+}	229.9	233.0	11476	9616		
	Mo^{6+}	232.5	235.5	18396	11044		
**890** ºC	Mo^{2+}	228.2	231.3	81689	57654	0.61	69.3
	Mo^{3+}	228.8	231.9	48914	35957		
	Mo^{4+}	229.9	233.0	10665	9307		
	Mo^{6+}	232.5	235.6	78555	53112		

Supplementary Table 7. Specific capacitance and ECSA for all HER electrodes in 0.5 M H_2SO_4.

**Electrode**	**C_{dl}(mF cm^{-2})**	**ECSA(cm$^2_{ECSA}$)**
**Mo/C-590**	5.68	142
**MoC-Mo_2C-690**	28.25	706
**MoC-Mo_2C -790**	111.12	2778
**Mo_2C -890**	34.12	853

5. Supplementary Figures

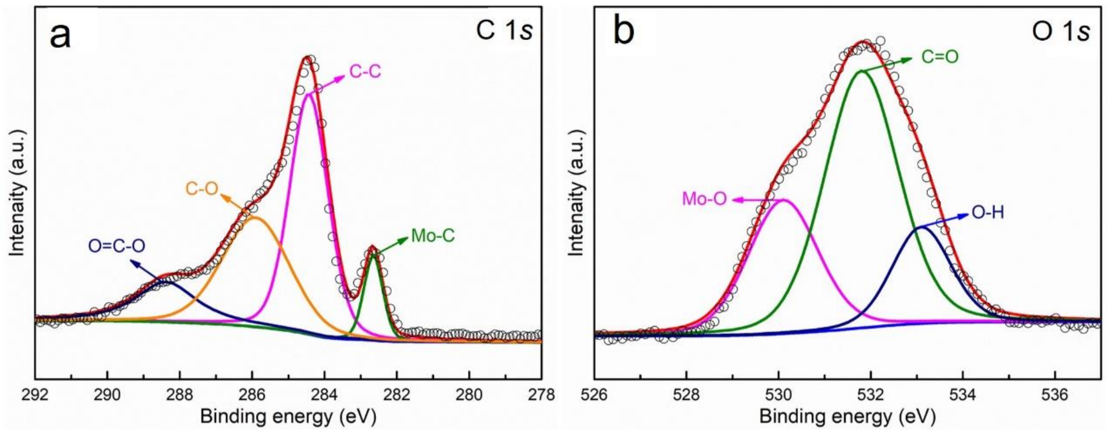

Supplementary Fig. 1 XPS spectrums of the MoC-Mo_2C-790 electrode. a C 1*s* XPS spectrum and O 1*s* XPS spectrum of the surface of the MoC-Mo_2C-790 electrode (**b**).

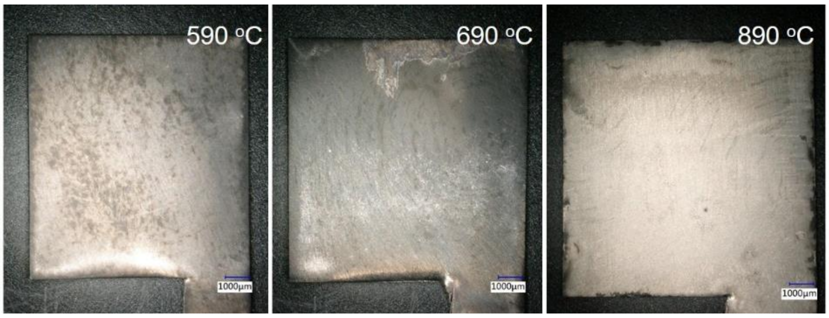

Supplementary Fig. 2 Digital photos of Mo and electrolytic electrodes prepared at 590 °C, 690 °C and 890 °C. The results show that temperature has a strong influence on the morphology of the electrode surface.

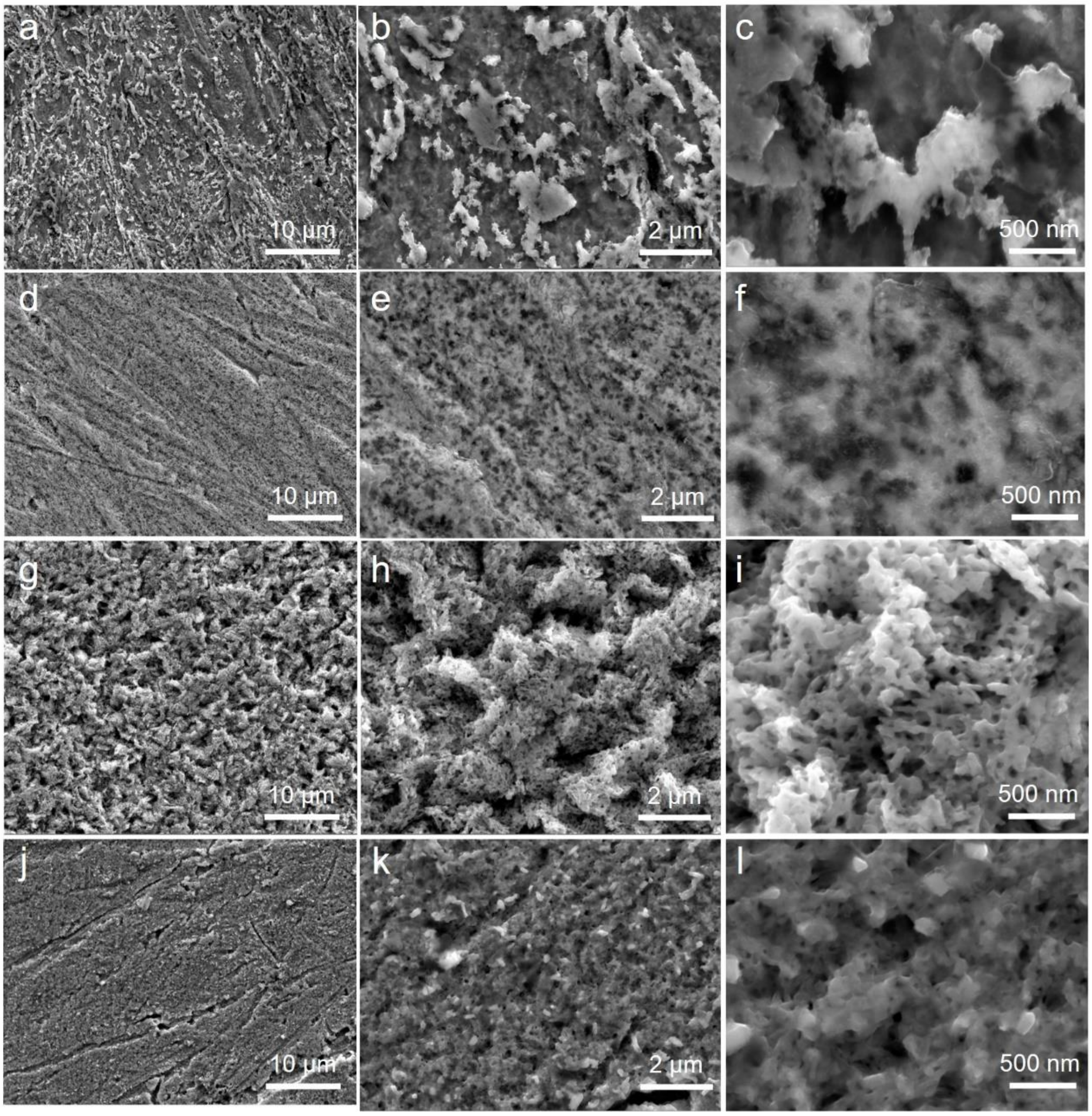

Supplementary Fig. 3 SEM images of different electrolytic electrodes prepared at 590 ºC (a-c), 690 ºC (d-f), 790 ºC (g-i) and 890 ºC (j-l). The results show that temperature has a strong influence on the micro-morphology of the electrode surface.

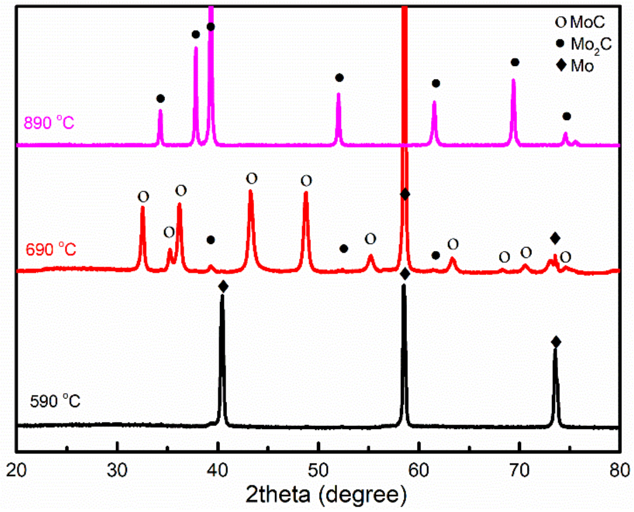

Supplementary Fig. 4 XRD patterns of different electrolytic electrodes prepared at 590 °C, 690 °C, and 890 °C. The results show that temperature has a strong influence on the composition of the electrode surface.

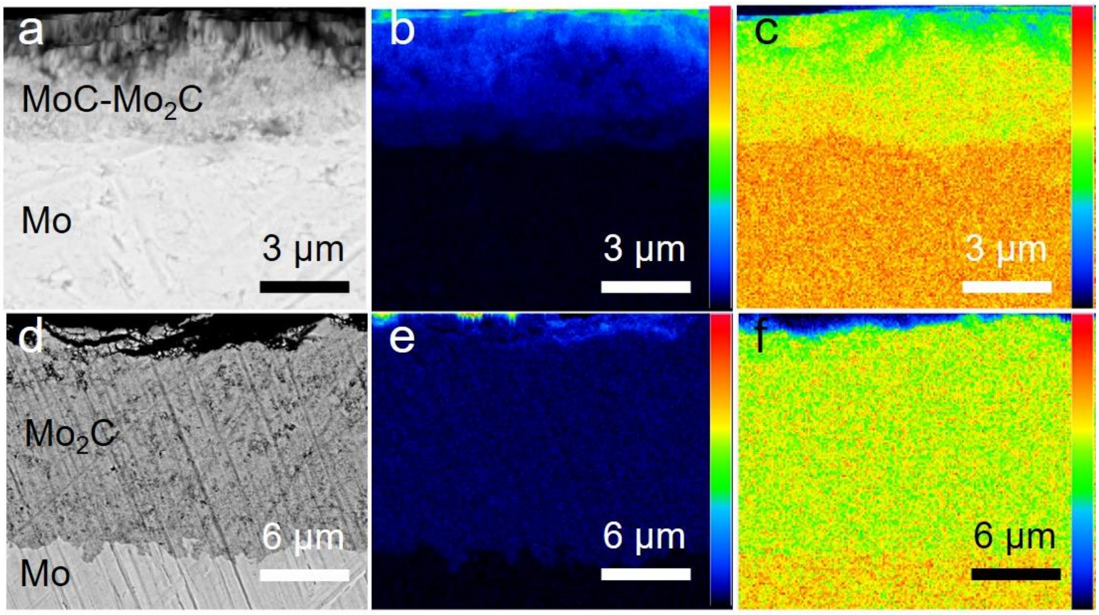

Supplementary Fig. 5 Cross-sectional element distribution mappings of different electrodes measured by EPMA. MoC-Mo_2C-690 (**a-c**) and Mo_2C-890 (**d-f**) electrodes. The results show that there are more MoC on the surface of the MoC-Mo_2C-690, and more Mo_2C at the bottom of MoC-Mo_2C-690. The Mo_2C-890 shows uniform distribution of Mo and C,

indicating the uniform Mo_2C composition.

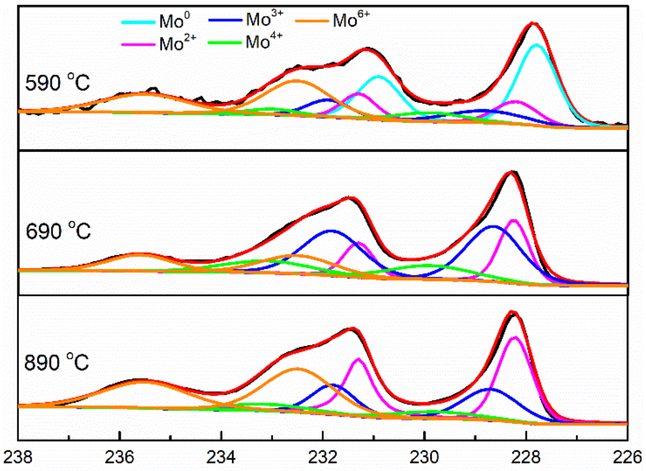

Supplementary Fig. 6 XPS patterns of different electrolytic electrodes prepared at 590 °C, 690 °C and 890 °C. The results show that temperature has a strong influence on the composition of the electrode surface.

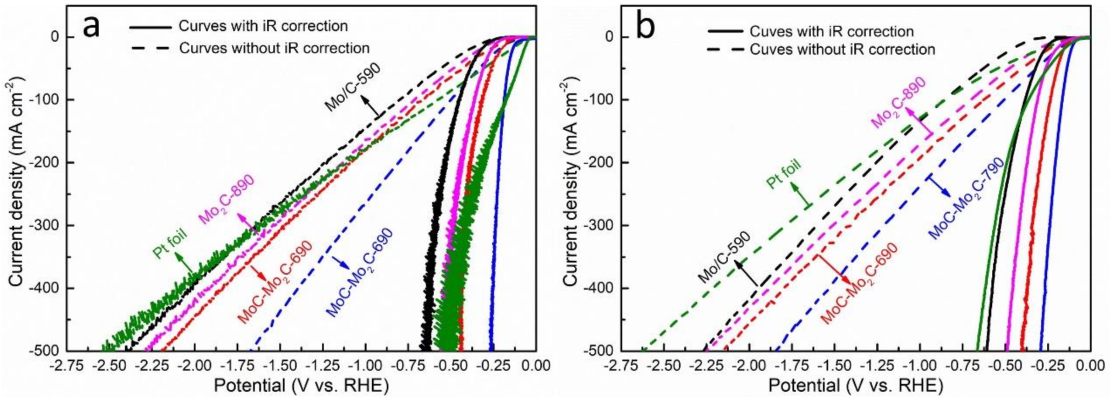

Supplementary Fig. 7 The LSV of different electrodes before and after iR correction in 0.5 M H_2SO_4 and 1 M KOH. a The LSV of different electrodes before and after iR correction in 0.5 M H_2SO_4. **b** The LSV of different electrodes before and after iR correction in 1.0 M KOH.

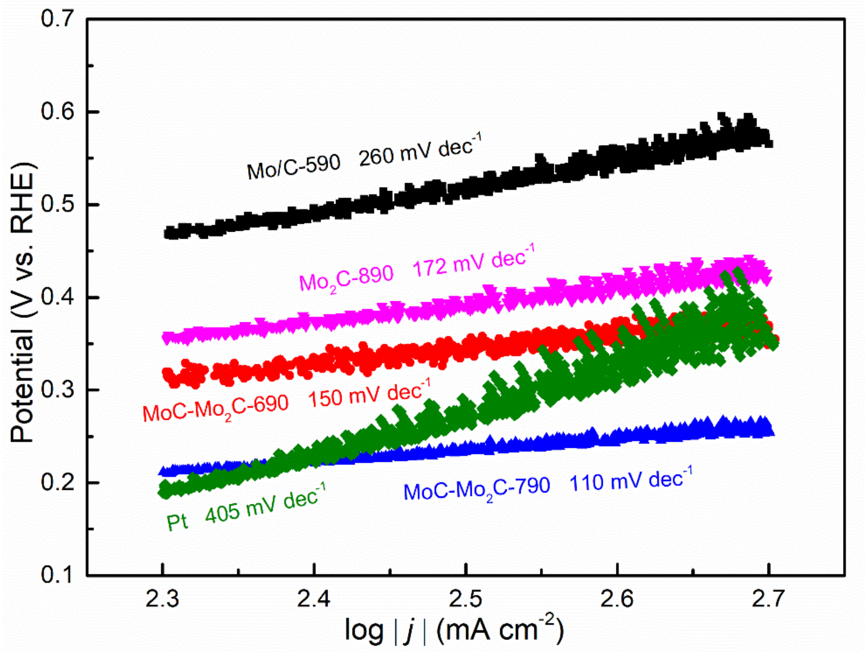

Supplementary Fig. 8 Slope plots of different electrodes at the current density ranging from 200 to 500 mA cm^{-2}. The result shows the needed potential when increasing the current to an industry-scale current, which could be an indicator to evaluate the performance of a catalyst at large current densities and is meaningful for practical use.

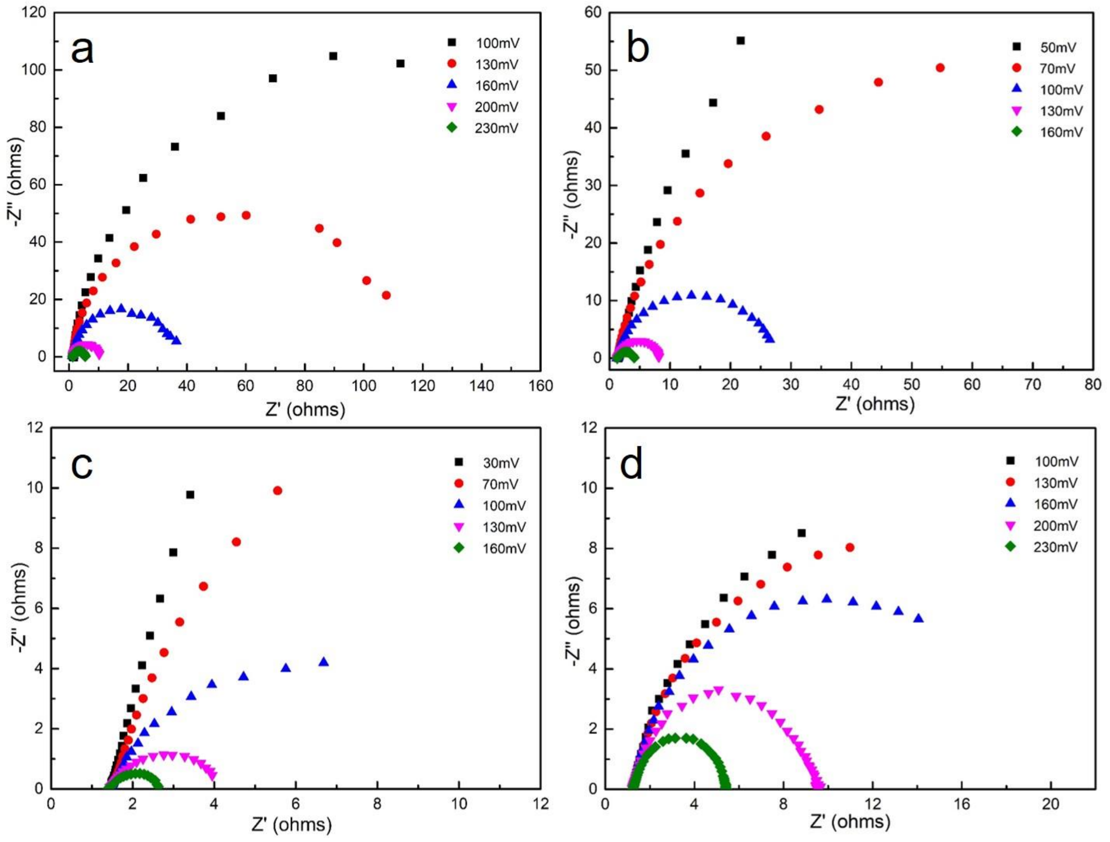

Supplementary Fig. 9 Nyquist plots of electrolytic electrodes prepared at different temperatures. a the Mo/C-590 electrode, **b** the MoC-Mo_2C-690 electrode, **c** the MoC-Mo_2C-790 electrode, **d** the Mo_2C-790 electrode.

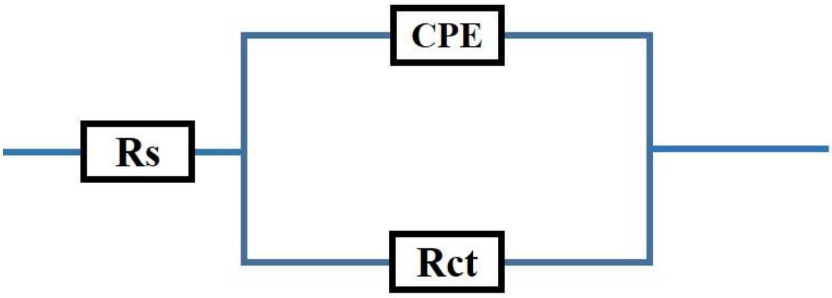

Supplementary Fig. 10 Equivalent circuit of the electrochemical impedance spectrum (EIS).

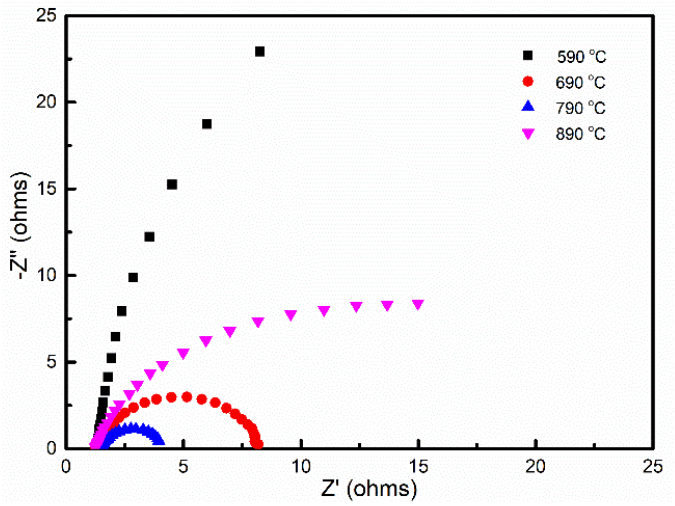

Supplementary Fig. 11 Nyquist plots of various electrodes prepared at different temperatures. The results show that the MoC-Mo_2C-790 electrode has the smallest charge transfer resistance.

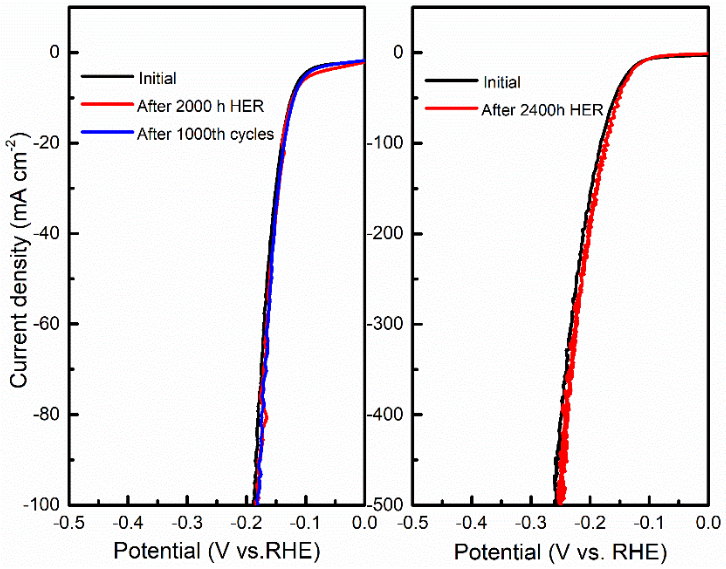

Supplementary Fig. 12 Polarization curves before and after 1000 CV cycles and 2000 h stability test, and polarization curves before and after 2400 h stability test. The results show that the MoC-Mo_2C electrode has a highly stability.

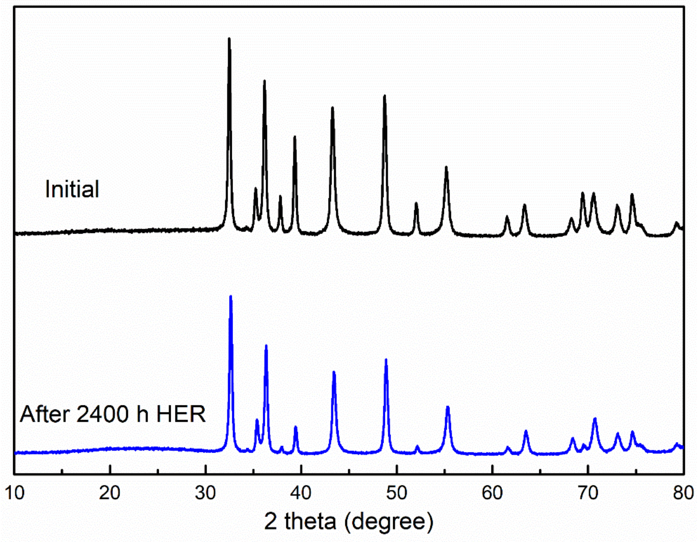

Supplementary Fig. 13 XRD patterns of the MoC-Mo_2C-790 electrode before and after 2400 h durability test. The results show that the MoC-Mo_2C electrode has a highly stability.

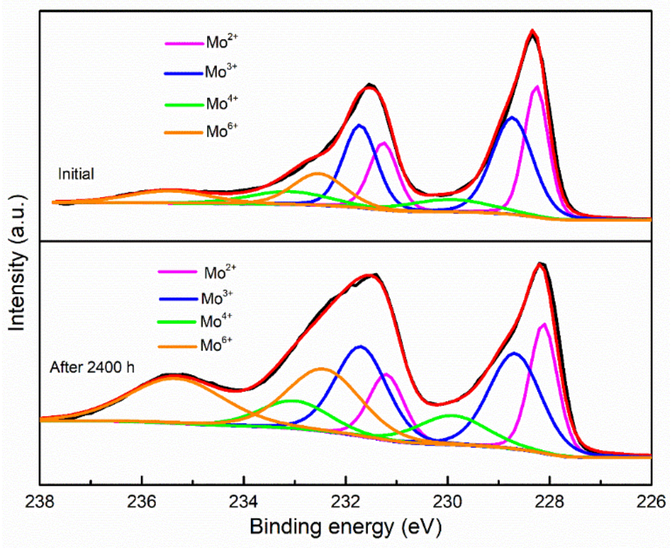

Supplementary Fig. 14 XPS patterns of the MoC-Mo_2C-790 electrode before and after 2400 h durability test. The almost unchanged XPS spectrums of Mo 3*d* before and after the stability test shows that the electrode has super stability.

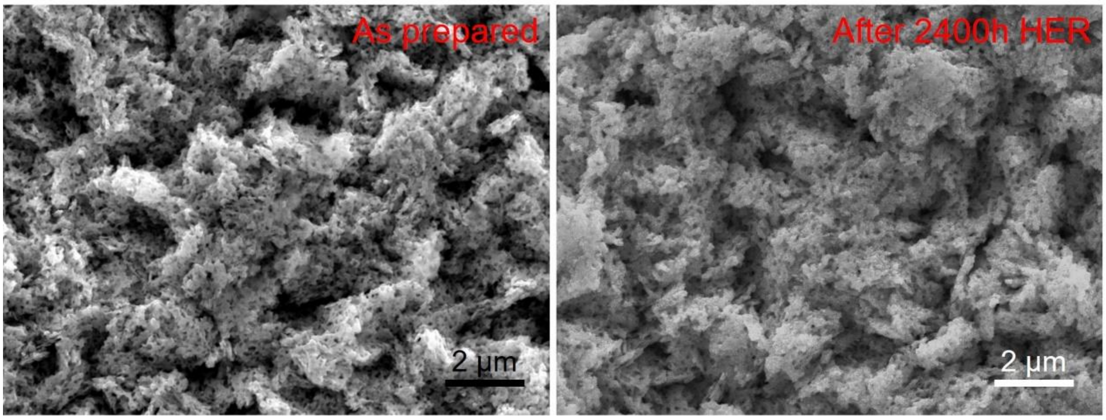

Supplementary Fig. 15 SEM images of the MoC-Mo_2C-790 electrode before and after 2400 h durability test. The almost unchanged SEM images of the MoC-Mo_2C-790 electrode before and after the stability test shows that the electrode has super stability.

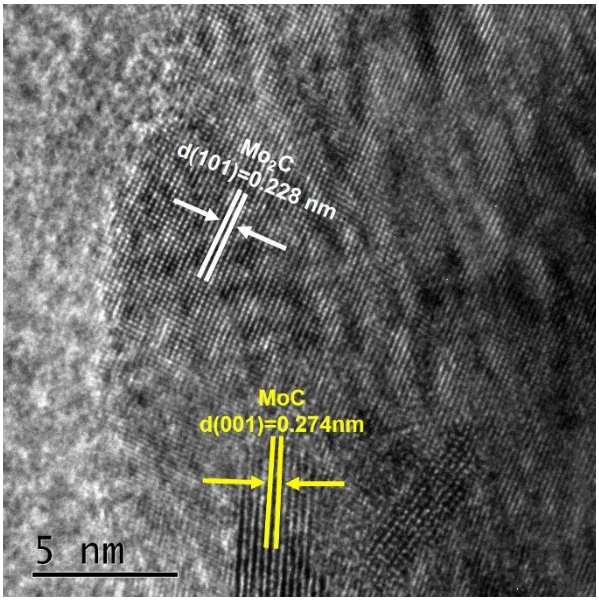

Supplementary Fig. 16 TEM images of the cross sectional MoC-Mo_2C-790 electrode after 2400 h's stability test. The almost unchanged TEM images of the MoC-Mo_2C-790 electrode before and after the stability test shows that the electrode has super stability.

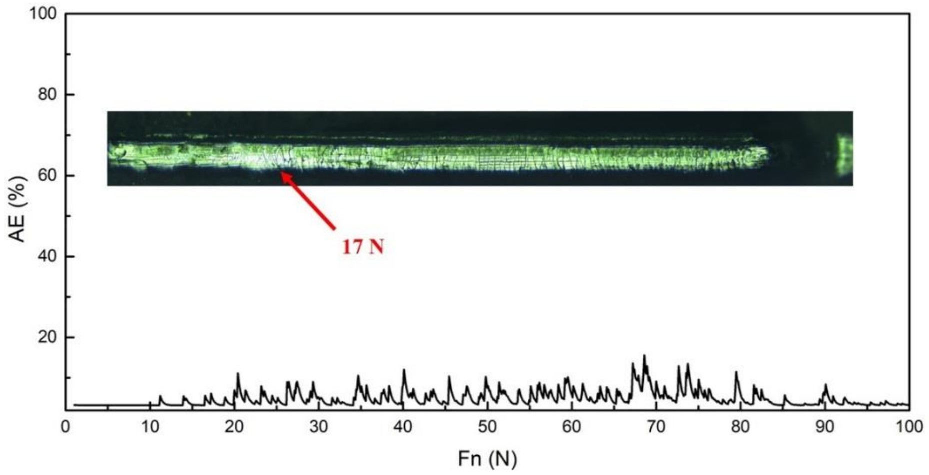

Supplementary Fig. 17 Binding test of the $MoC\text{-}Mo_2C$ film on the $MoC\text{-}Mo_2C$-790 electrode. The binding test of the $MoC\text{-}Mo_2C$ film indicates that the active materials on the surface of the $MoC\text{-}Mo_2C$-790 electrode has a strong binding force, which will help improve the corresponding HER stability.

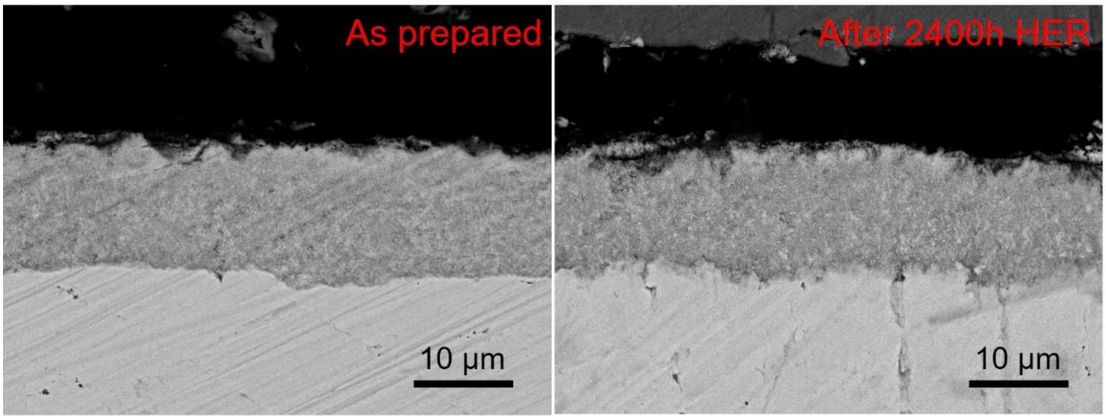

Supplementary Fig. 18 SEM images of the cross sectional of the $MoC\text{-}Mo_2C$-790 electrode before and after 2400 h stability test. The almost unchanged cross-sectional SEM images of the $MoC\text{-}Mo_2C$-790 electrode before and after the stability test shows that the electrode has super stability.

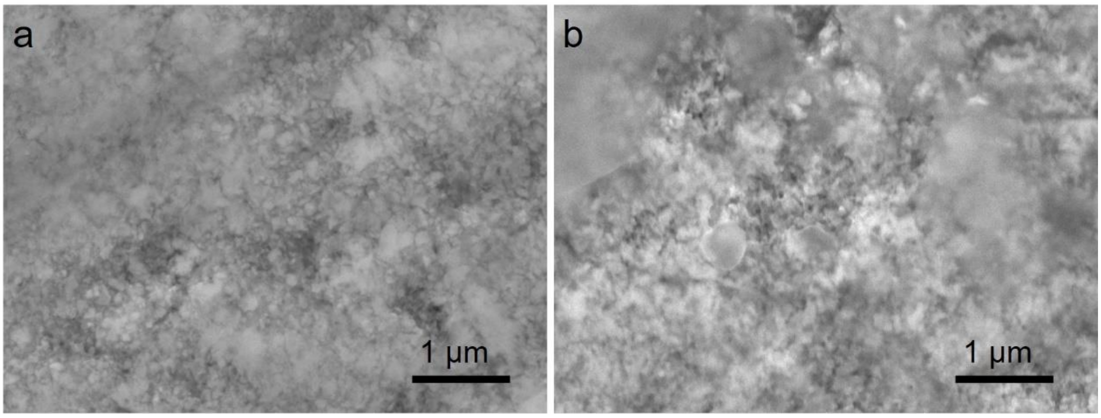

Supplementary Fig. 19 SEM images at large multiple of cross section of the MoC-Mo_2C-790 electrode before (a) and after (b) 2400 h stability test. The results show that the MoC-Mo_2C coating is uniform and dense, which is beneficial to the improvement of HER stability.

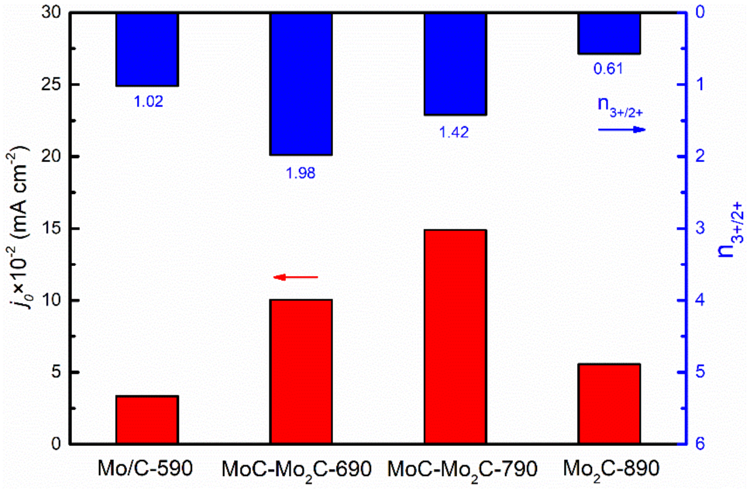

Supplementary Fig. 20 Relationship between the exchange current density (*j_0*) of HER electrodes and the ratio of surface Mo^{3+}/Mo^{2+} calculated from XPS analysis. The results indicate that the different **Mo^{3+}/Mo^{2+}** radio is able to affect the HER performance of the electrodes.

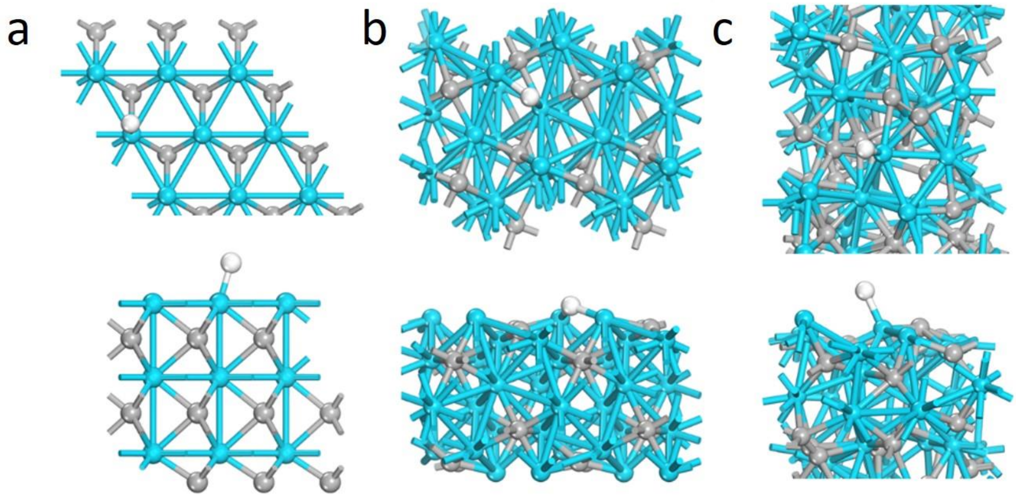

Supplementary Fig. 21 Different views of the optimized structural model. Top and side views of (**a**) MoC and (**b**) Mo_2C, and (**c**) MoC-Mo_2C with H adatom.

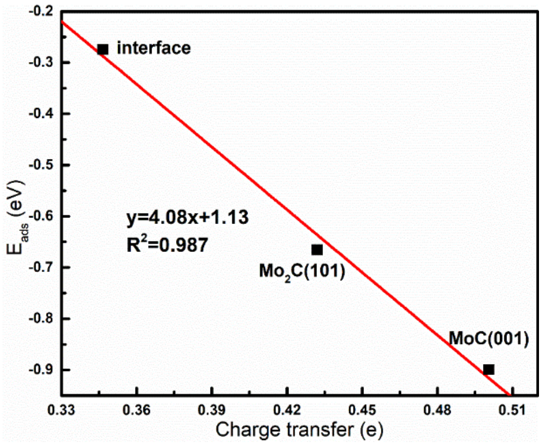

Supplementary Fig. 22 The correlation of atomic H adsorption energy with the charge transfer. R^2, coefficient of determination.

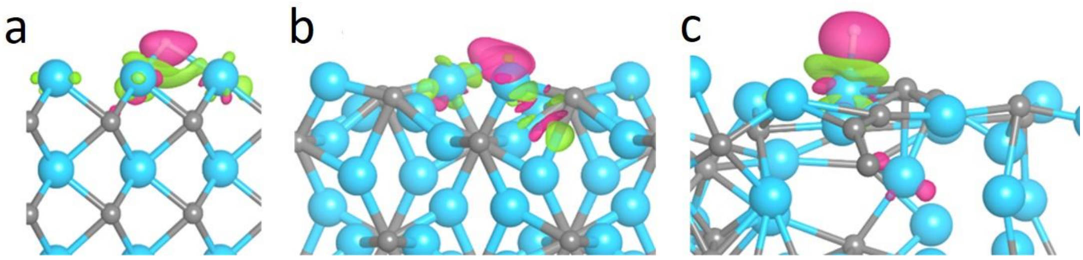

Supplementary Fig. 23 Charge density difference for (a) MoC, (b) Mo2C, and (c) MoC-Mo2C heterojunction interface with the H adatom. The pink and green regions represent electron accumulation and depletion, respectively.

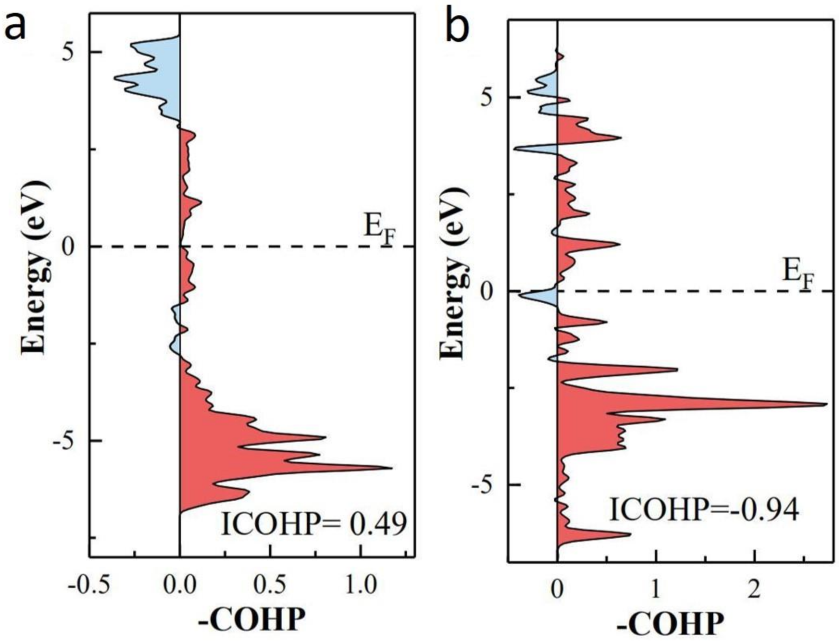

Supplementary Fig. 24 Projected COHP for H adsorbed (a) Mo_2C, and (b) MoC-Mo_2C heterojunction interface. The bonding (red) and antibonding (cyan) contributions are displayed on the right panel and left panel, respectively.

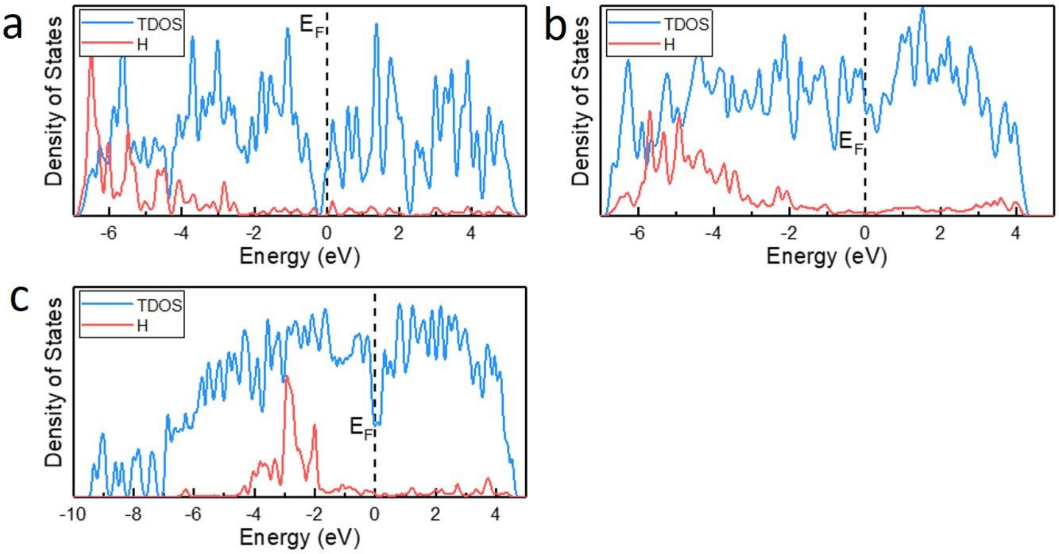

Supplementary Fig. 25 PDOS of (a) MoC, (b) Mo_2C, and (c) MoC-Mo_2C heterojunction interface.

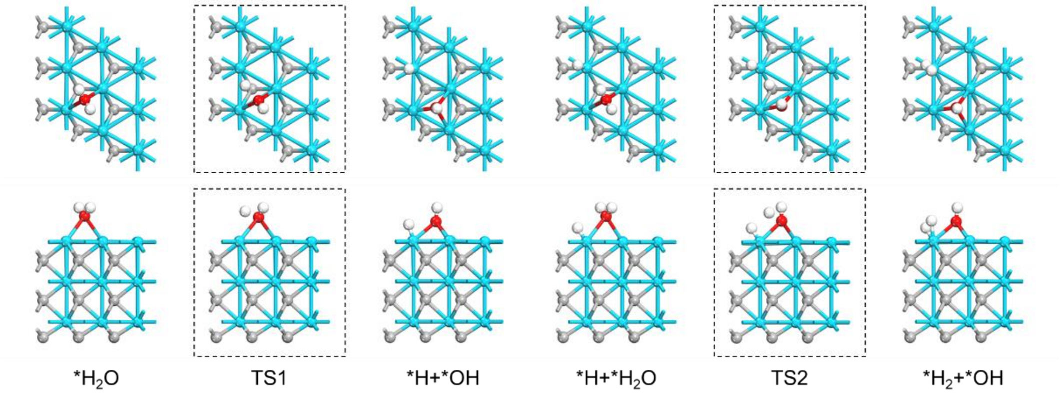

Supplementary Fig. 26 The top-view and side-view structures of intermediates on the bare MoC during the process of alkaline HER.

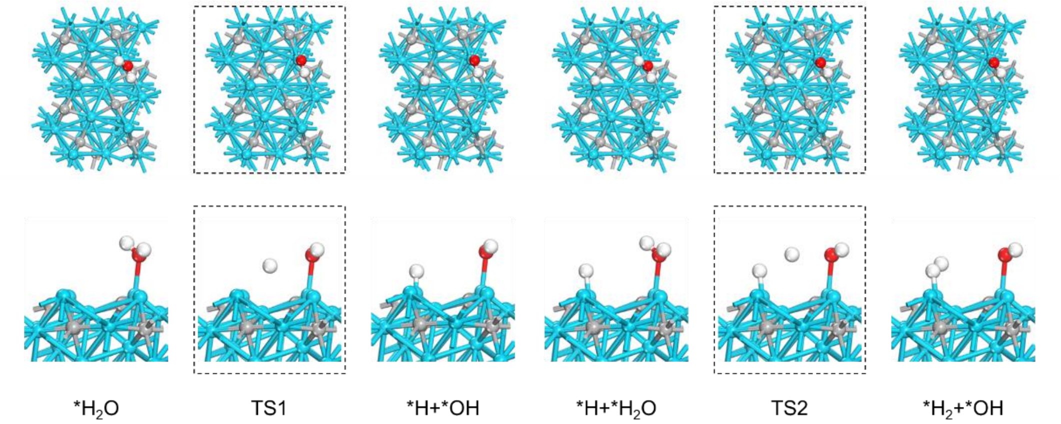

Supplementary Fig. 27 The top-view and side-view structures of intermediates on the bare Mo_2C during the process of alkaline HER.

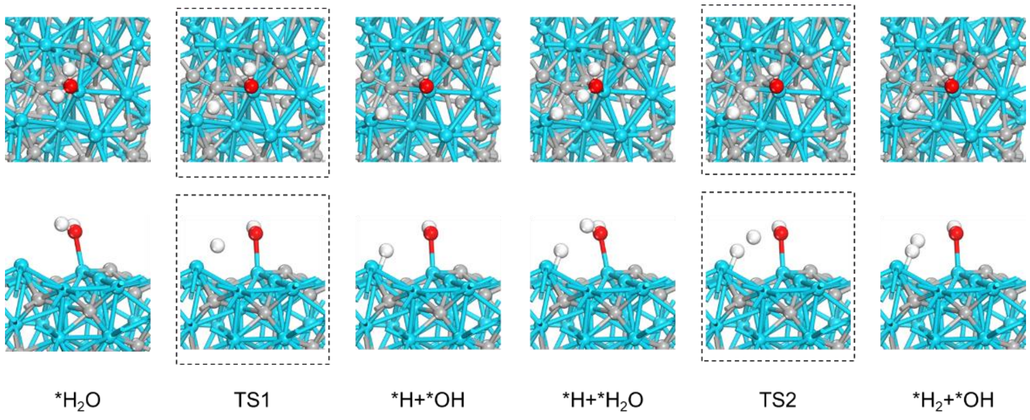

Supplementary Fig. 28 The top-view and side-view structures of intermediates on the $MoC-Mo_2C$ during the process of alkaline HER.

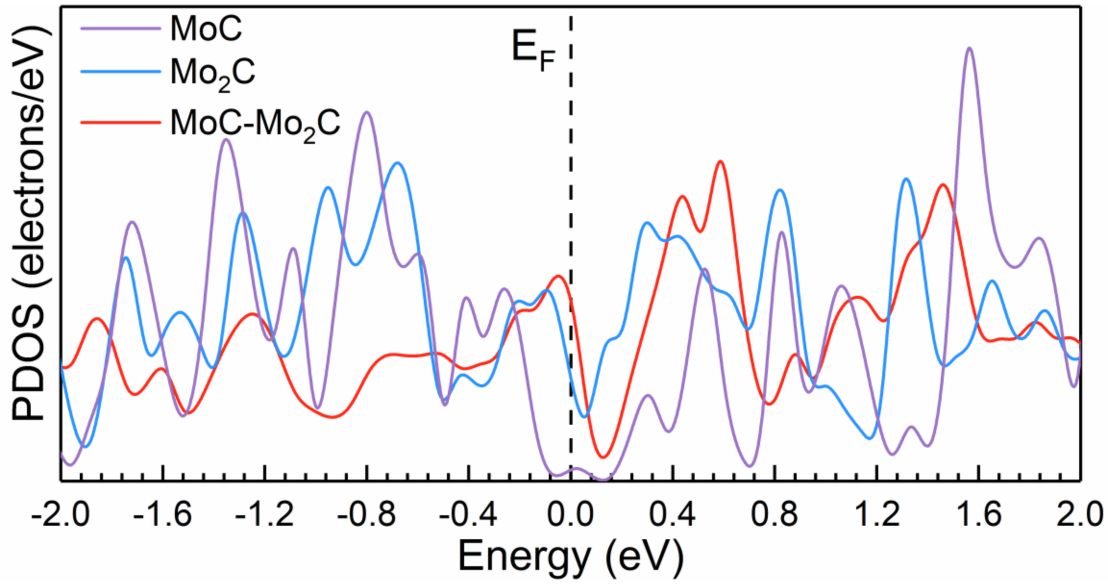

Supplementary Fig. 29 Partial density of states of Mo d states for MoC, Mo_2C, and $MoC-Mo_2C$.

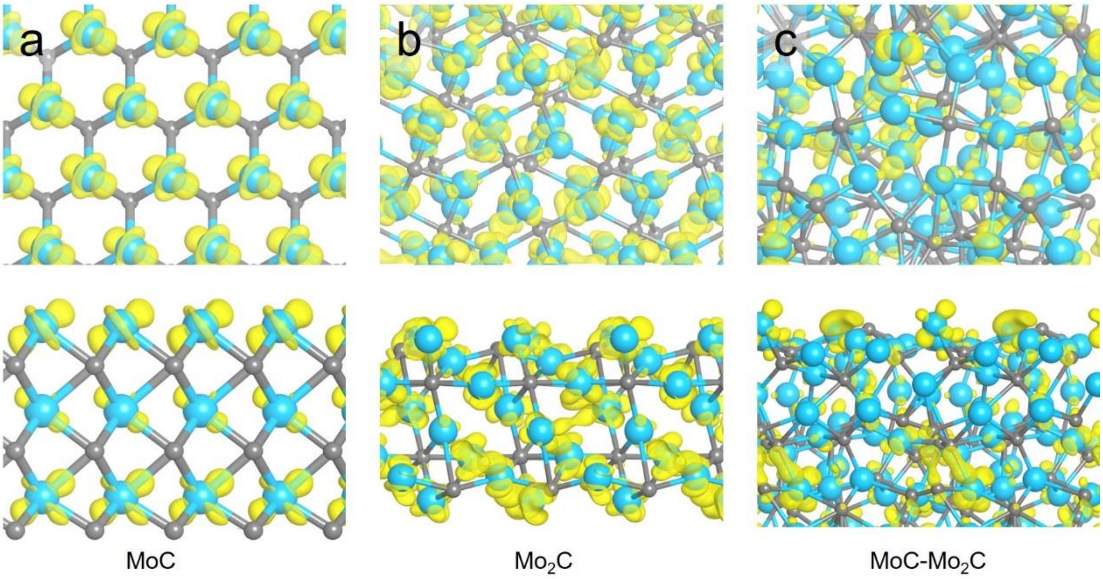

Supplementary Fig. 30 Partial charge density for (a) MoC, (b) Mo_2C, and (c) MoC-Mo_2C heterojunction interface. The yellow region represents electron accumulation.

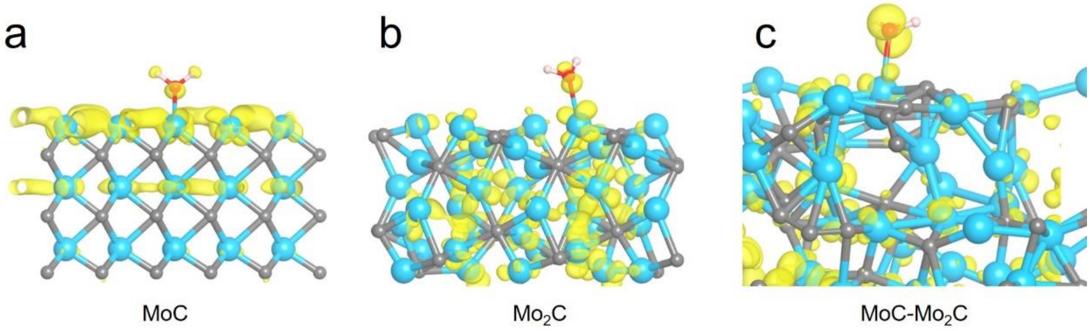

Supplementary Fig. 31 Partial charge density for (a) MoC, (b) Mo_2C, and (c) MoC-Mo_2C heterojunction interface with the adsorbed H_2O. The yellow region represents electron accumulation.

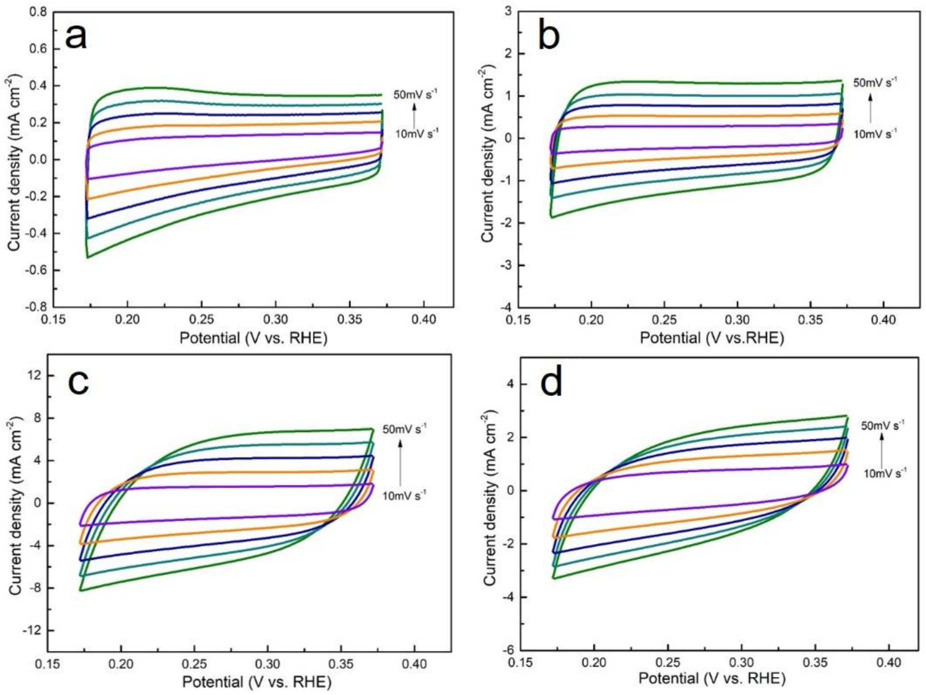

Supplementary Fig. 32 Cyclic voltammograms of different electrolytic electrodes. a the Mo/C-590 electrode, **b** the MoC-Mo_2C-690 electrode, **c** the MoC-Mo_2C-790 and (**d**) the Mo_2C-890 electrode.

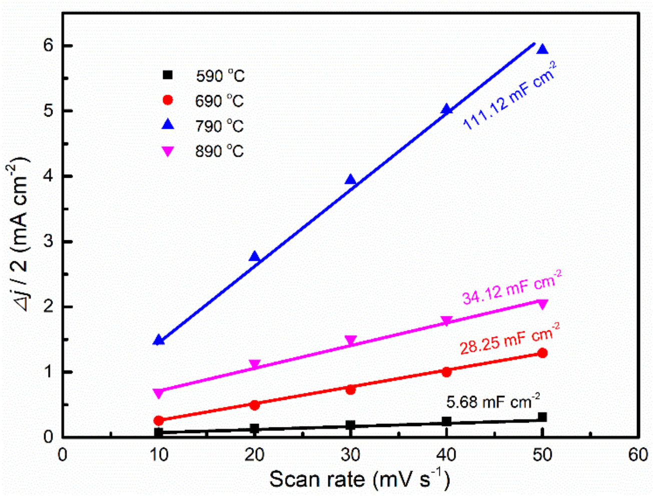

Supplementary Fig. 33 Estimation of C_{dl} by plotting capacitive current density and the scan rate of various self-supported electrode in non-faradaic regions. The results show that the MoC-Mo_2C-790 has the largest electrochemical surface area.

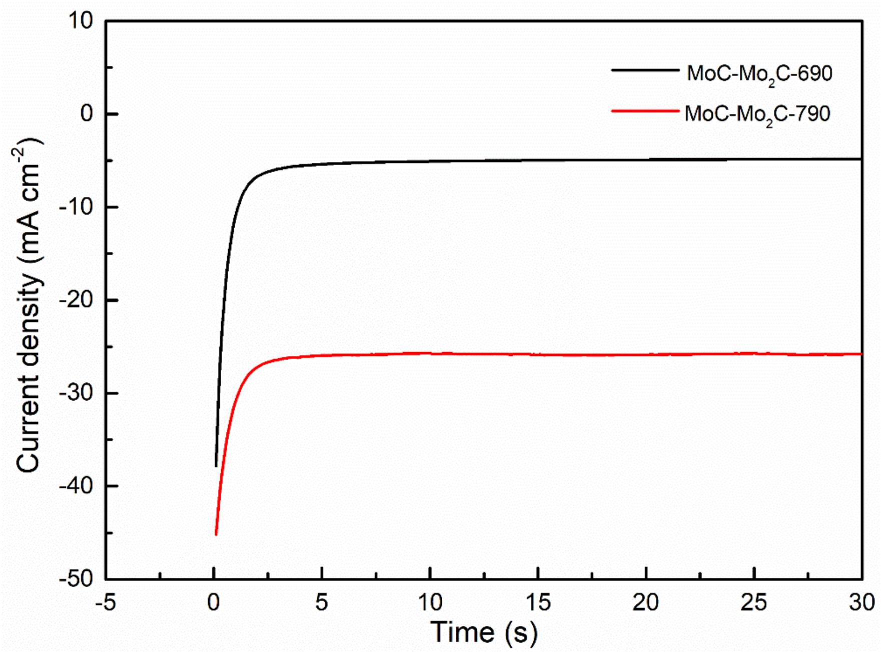

Supplementary Fig. 34 I-t curves of electrolytic electrodes prepared at 690 ºC and 790 ºC at the constant overpotential of 140 mV. The results the MoC-Mo_2C-690 is affected more highly by the shielding effect of bubbles on the electrode surface.

6. Supplementary References

S1. Li, Y. et al. 3D Self-Supported Fe-Doped Ni_2P Nanosheet Arrays as Bifunctional Catalysts for Overall Water Splitting. *Adv. Funct. Mater.* **27**, 1702513 (2017).

S2. Yu, S. H. et al. Highly stable tungsten disulfide supported on a self-standing nickel phosphide foam as a hybrid electrocatalyst for efficient electrolytic hydrogen evolution. *Nano Energy* **55**, 193-202 (2019).

S3. Zhang, Q. et al. Myriophyllum-like hierarchical TiN@Ni_3N nanowire arrays for bifunctional water splitting catalysts. *J. Mater. Chem. A* **4**, 5713-5718 (2016).

S3. Xiao, J. et al. Self-supported biocarbon-fiber electrode decorated with molybdenum carbide nanoparticles for highly active hydrogen-evolution reaction. *ACS Appl. Mater. Interfaces* **9**, 22604-22611 (2017).

S5. Jia, Y. et al. A heterostructure coupling of exfoliated Ni-Fe hydroxide nanosheet and defective graphene as a bifunctional electrocatalyst for overall water splitting. *Adv. Mater.* **29**, 1700017 (2017).

S6. Ekspong, J. et al. Stabilizing Active Edge Sites in Semicrystalline Molybdenum Sulfide by Anchorage on Nitrogen-Doped Carbon Nanotubes for Hydrogen Evolution Reaction. *Adv. Funct. Mater.* **26**, 6766-6776 (2016).

S7. Wu, X. et al. Engineering Multifunctional Collaborative Catalytic Interface Enabling Efficient Hydrogen Evolution in All pH Range and Seawater. *Adv. Energy Mater.* **9**, 1901333 (2019).

S8. Bose, R. et al. Molybdenum sulphoselenophosphide spheroids as an effective catalyst for hydrogen evolution reaction. *Small*, **14**, 1703862 (2018).

S9. Jiao, S. et al. Defect-rich one-dimensional MoS_2 hierarchical architecture for efficient hydrogen evolution: Coupling of multiple advantages into one catalyst. *Appl. Catal., B* **258**, 117964 (2019).

S10. Wang, H. et al. Optimizing MoS_2 edges by alloying isovalent W for robust hydrogen evolution activity. *ACS Catal.* **8**, 9529-9536 (2018).

S11. Zhu, J. et al. Boundary activated hydrogen evolution reaction on monolayer MoS_2. *Nat. Commun.* **10**, 1348 (2019).

S12. Wang, H. et al. Bifunctional non-noble metal oxide nanoparticle electrocatalysts through lithium-induced conversion for overall water splitting. *Nat. Commun.* **6**, 7261 (2015).

S13. Li, Y. et al. Nickel-molybdenum nitride nanoplate electrocatalysts for concurrent electrolytic hydrogen and formate productions. *Nat. Commun.* **10**, 1-12 (2019).

S14. Wu, Y. et al. Electron density modulation of $NiCo_2S_4$ nanowires by nitrogen incorporation for highly efficient hydrogen evolution catalysis. *Nat. Commun.* **9**, 1425 (2018).

S15. Chen, Y. C. et al. Structurally deformed MoS_2 for electrochemically stable, thermally resistant, and highly efficient hydrogen evolution reaction. *Adv. Mater.* **29**, 1703863 (2017).

S16. Liu, B. et al. Few Layered N, P Dual-Doped Carbon-Encapsulated Ultrafine MoP Nanocrystal/MoP Cluster Hybrids on Carbon Cloth: An Ultrahigh Active and Durable 3D Self-Supported Integrated Electrode for Hydrogen Evolution Reaction in a Wide pH Range. *Adv. Funct. Mater.* **28**, 1801527 (2018).

S17. Huang, L. B. et al. Self-limited on-site conversion of MoO_3 nanodots into vertically aligned ultrasmall monolayer MoS_2 for efficient hydrogen evolution. *Adv. Energy Mater.* **8**, 1800734 (2018).

S18. Jia, J. et al. Molybdenum carbide on hierarchical porous carbon synthesized from Cu-MoO_2 as efficient electrocatalysts for electrochemical hydrogen generation. *Nano Energy* **41**, 749-757 (2017).

S19. Li, L. et al. P-Doped MoO_{3-x} Nanosheets as Efficient and Stable Electrocatalysts for Hydrogen Evolution. *Small* **13**, 1700441 (2017).

S20. Yan, Y. et al. Vertically oriented MoS_2 and WS_2 nanosheets directly grown on carbon cloth as efficient and stable 3-dimensional hydrogen-evolving cathodes. *J. Mater. Chem. A* **3**, 131-135 (2015).

S21. Chen, Y. C. et al. Structurally deformed MoS_2 for electrochemically stable, thermally resistant, and highly efficient hydrogen evolution reaction. *Adv. Mater.* **29**, 1703863 (2017).

S22. Liu, B. et al. Few Layered N, P Dual-Doped Carbon-Encapsulated Ultrafine MoP Nanocrystal/MoP Cluster Hybrids on Carbon Cloth: An Ultrahigh Active and Durable 3D Self-Supported Integrated Electrode for Hydrogen Evolution Reaction in a Wide pH Range. *Adv. Funct. Mater.* **28**, 1801527 (2018).

S23. Xing, Z. et al. Closely interconnected network of molybdenum phosphide nanoparticles: a highly efficient electrocatalyst for generating hydrogen from water. *Adv. Mater.* **26**, 5702-5707 (2014).

S24. Chen, Y. et al. Highly active, nonprecious electrocatalyst comprising borophene subunits for the hydrogen evolution reaction. *J. Am. Chem. Soc.* **139**, 12370-12373 (2017).

S25. Duan, J. et al. Porous C_3N_4 nanolayers@ N-graphene films as catalyst electrodes for highly efficient hydrogen evolution. *ACS nano* **9**, 931-940 (2015).

S26. Deng, J. et al. Multiscale structural and electronic control of molybdenum disulfide foam

for highly efficient hydrogen production. *Nat. Commun.* **8**, 14430 (2017).

S27. Jiao, S. et al. Defect-rich one-dimensional MoS_2 hierarchical architecture for efficient hydrogen evolution: Coupling of multiple advantages into one catalyst. *Appl. Catal., B* **258**, 117964 (2019).

S28. He, C. et al. Complex vectorial optics through gradient index lens cascades. Nat. Commun. 10, 1-8 (2019).

S29. Yan, H. et al. Cluster-like molybdenum phosphide anchored on reduced graphene oxide for efficient hydrogen evolution over a broad pH range. *Chem. Commun.* **52**, 9530-9533 (2016).

S30. Li, Y. et al. 3D Self-Supported Fe-Doped Ni_2P Nanosheet Arrays as Bifunctional Catalysts for Overall Water Splitting. *Adv. Funct. Mater.* **27**, 1702513 (2017).

S31. Liu, H. et al. Heteromorphic $NiCo_2S_4/Ni_3S_2/Ni$ foam as a self-standing electrode for hydrogen evolution reaction in alkaline solution. *ACS Appl. Mater. Interfaces* **10**, 10890-10897 (2018).

S32. Sheng, M. et al. Network-like porous Co-Ni-B grown on carbon cloth as efficient and stable catalytic electrodes for hydrogen evolution. *Electrochem. Commun.* **93**, 104-108 (2018).

S33. Pu, Z. et al. Semimetallic MoP_2: an active and stable hydrogen evolution electrocatalyst over the whole pH range. *Nanoscale* **8**, 8500-8504 (2016).

S34. Wu, Z. et al. Facile preparation of carbon sphere supported molybdenum compounds (P, C and S) as hydrogen evolution electrocatalysts in acid and alkaline electrolytes. *Nano Energy* **32**, 511-519 (2017).

S35. Zhang, T. et al. Engineering oxygen vacancy on NiO nanorod arrays for alkaline hydrogen evolution. *Nano Energy* **43**, 103-109 (2018).

S36. Wang, H. et al. Bifunctional non-noble metal oxide nanoparticle electrocatalysts through lithium-induced conversion for overall water splitting. *Nat. commu.* **6**, 7261 (2015).

S37. Wang, X. et al. Hierarchically Porous W-Doped CoP Nanoflake Arrays as Highly Efficient and Stable Electrocatalyst for pH-Universal Hydrogen Evolution. *Small* (2019).

S38. Zhuang, Z. et al. MoB/g-C_3N_4 Interface Materials as a Schottky Catalyst to Boost Hydrogen Evolution. *Angew. Chem. Int. Ed.* **57**, 496-500 (2018).

S39. Meng, X. et al. Three-dimensionally hierarchical MoS_2/graphene architecture for high-performance hydrogen evolution reaction. *Nano Energy* **61**, 611-616 (2019).

S40. Majee, R. et al. Tweaking Nickel with Minimal Silver in a Heterogeneous Alloy of Decahedral Geometry to Deliver Platinum-like Hydrogen Evolution Activity. *Angew. Chem. Int. Ed.* (2019).

S41. Kibsgaard, J. et al. Designing an improved transition metal phosphide catalyst for hydrogen evolution using experimental and theoretical trends. *Energy Environ. Sci.* **8**, 3022-3029 (2015).

S42. Kibsgaard, J & Jaramillo, T. F. Molybdenum phosphosulfide: an active, acid-stable, earth-abundant catalyst for the hydrogen evolution reaction. *Angew. Chem. Int. Ed.* 53, 14433-14437 (2014).

S43. Xue, et al. Rationally engineered active sites for efficient and durable hydrogen generation. *Nat. commu.* **10**, 2281 (2019).

S44. Khani, H. et al. Graphitic-Shell Encapsulation of Metal Electrocatalysts for Oxygen Evolution, Oxygen Reduction, and Hydrogen Evolution in Alkaline Solution. *Adv. Energy Mater.* (2019).

S45. Wang Z. et al. Copper-Nickel Nitride Nanosheets as Efficient Bifunctional Catalysts for Hydrazine-Assisted Electrolytic Hydrogen Production. *Adv. Energy Mater.* 1900390 (2019).

S46. Xu Q. et al. Unsaturated sulfur edge engineering of strongly coupled MoS_2 nanosheet-carbon macroporous hybrid catalyst for enhanced hydrogen generation. *Adv. Energy Mater.* 9, 1802553 (2019).
