# Supplementary material for: A durable and pH-universal self-standing MoC–Mo2C heterojunction electrode for efficient hydrogen evolution reaction
Source: Nat Commun. 2021 Nov 22;12:6776. doi: 10.1038/s41467-021-27118-6 (PMC8608917; doi:10.1038/s41467-021-27118-6)
Supplement: Supplementary file 3 — Description of Additional Supplementary Files [file 41467_2021_27118_MOESM3_ESM.pdf]

Description of Additional Supplementary Files

Supplementary Movie 1:

The HER process of Mo/C-590 in 0.5 M H2SO4 at the current density of 40 mA cm-2 .

Supplementary Movie 2:

The HER process of MoC-Mo2C-690 in 0.5 M H2SO4 at the current density of 40 mA cm-2 .

Supplementary Movie 3:

The HER process of MoC-Mo2C-790 in 0.5 M H2SO4 at the current density of 40 mA cm-2 .

Supplementary Movie 4:

The HER process of Mo2C-790 in 0.5 M H2SO4 at the current density of 40 mA cm-2 .

Supplementary Movie 5:

The HER process of Pt foil in 0.5 M H2SO4 at the current density of 40 mA cm-2 .

Supplementary Movie 6:

The HER process of the enlarged MoC-Mo2C790 electrode in 0.5 M H2SO4 at the current density of 40 mA cm
